# 

**Published:** 1836-10

**Authors:** 


### British and Foreign Medical Review.

No. IV.

### NOTICES to CONTRIBUTORS, CORRESPONDENTS, and SUBSCRIBERS.

1. *The Editors regret that, notwithstanding the present Number contains no less than two sheets of letter-press beyond the quantity originally announced, they are still compelled, by want of room, to postpone several important communications from their Contributors. Indeed, such is the continued pressure of valuable matter, that they have it in contemplation, by a slight change in the First Department, to render the already ample space of the Review still more capacious: of course, without any increase of the price.*

2. *The author of the Article on the recent Work of Bouillaud is respectfully informed, that it is not required for the British and Foreign Medical Review; and he may have the Paper on application to the Publishers.*

3. *All Communications, Books, and Letters on the subject of the Review, are to be addressed to the Editors, care of the Publishers, Paternoster Row; and Gentlemen residing in the Colonies or in Foreign Countries, are requested to take care, in transmitting Contributions and Books for Review, to render the postage and carriage as small as may be practicable: this is sometimes most disproportionately large.*

### BOOKS RECEIVED FOR REVIEW.

1. Elements of Medical Jurisprudence. By T. R. Beck, m.d., Professor of the Institutes of Medicine, &c., and J. B. Beck, m.d., Professor of Materia Medica, &c. Fifth Edition.—Two vols. 8vo. pp. 661. 694. Albany, 1835.

2. A Course of Legal Study. By David Hoffmann, Sur. Utr. Doct. Göttingen. Second Edition, rewritten and much enlarged. — Two volumes. 8vo. pp. 876. Baltimore, 1836.

3. The Philosophy of Living; or, the Way to enjoy Life and its Comforts. By Caleb Ticknor, a.m. m.d. — New York, 1836. 12mo. pp. 334.

4. Elements of Medical Jurisprudence. By A. S. Taylor, Esq., Lecturer on Medical Jurisprudence, &c. in Guy's Hospital. Vol. I.—London, 1836. 8vo. pp. 511.

5. A Dictionary of Terms employed by the French in Anatomy, Physiology, Pathology, &c.; with their Derivations, Synonyms, &c. Part II. By Shirley Palmer, m.d.—London, 1836. 8vo. pp. 160. (Cor—Hui.)

6. Aur. Cor. Celsus on Medicine, in eight Books, Latin and English. Translated from L. Targa's Edition, the Words of the Text being arranged in the order of Construction. To which are prefixed, a Life of the Author, Tables of Weights and Measures, with explanatory Notes, &c., designed to facilitate the Progress of Medical Students. By Alexander Lee, a.m. Surgeon.—Two vols. 8vo. pp. 318, 501. London, 1831, 1836.

7. The Cotton Manufacture of Great Britain systematically investigated, and illustrated by 150 original Figures, engraved on wood and steel; with an introductory View of its comparative State in Foreign Countries, drawn chiefly from Personal Survey. By Andrew Ure, m.d. f.r.s. &c.—London, 1836. Two vols. 8vo. pp. 359, 455.

8. The Proofs of Infanticide considered; including Dr. Hunter's Tract on Child-murder, with illustrative Notes; and a Summary of the present State of Medico-legal Knowledge on that subject. By Wm. Cummin, m.d., Lecturer on Forensic Medicine at the Aldersgate School.—London, 1836. 12mo. pp. 95.

9. The Speculum applied to the Diagnosis and Treatment of the Organic Diseases of the Womb: an Inaugural Dissertation presented to the University of Glasgow for the Degree of Doctor in Medicine. By John Balbirnie, a.m.—London, 1836. 8vo. pp. 335.

10. A Treatise on some Nervous Disorders; being chiefly intended to illustrate those Varieties which simulate Structural Disease. By Edwin Lee, m.r.c.s.—London, 1835. 8vo. pp. 152.

11. On Deformities of the Chest. By Wm. Coulson, Consulting Surgeon to the London Lying-in Hospital, &c.—London, 1836. 8vo. pp. 71; with Plates.

12. Report of the Directors of the Dundee Lunatic Asylum, for the year ending 31st May, 1836.—Dundee, 1836. 8vo. pp. 37.

13. On the Efficacy of Carbonic Acid Gas in the Diseases of Tropical Climates; with Directions for the Treatment of the Acute and Chronic Stages of Dysentery By John Parkin, Member of the Royal College of Surgeons, &c.—London, 1836. 8vo. pp. 64.

14. A few Hints on the Principle of Self-supporting Dispensaries, and its Adaptation to the Medical Relief of the Poor. By Charles Wilkinson, M.D.—London, 1836. 8vo. pp. 47.

15. The Practical Anatomy and Elementary Physiology of the Nervous System; designed for the use of Students in the Dissecting Room. By F. Le Gros Clark, Demonstrator of Anatomy in St. Thomas's Hospital.—London, 1836. 8vo. pp. 367.

16. An Introductory Lecture delivered to the Mathematical Class at the Royal School of Medicine and Surgery at Birmingham, 12th May, 1836. By the Rev. W. M. Lawson, M.A.—Birmingham, 1836. 8vo. pp. 53.

17. The Medical Works of Paulus Ægineta, translated into English, with a copious Commentary. Vol. I. By Francis Adams, Esq., Surgeon. — London and Aberdeen, 1834. 8vo. pp. 474.

18. Researches on the Effects of Blood-letting in some Inflammatory Diseases, and on the Influence of Tartarized Antimony and Vesication in Pneumonitis. By P. C. A. Louis. Translated by C. G. Putnam, M.D. With a Preface and Appendix, by James Jackson, M.D., Physician of the Massachussets General Hospital.—Boston, 1836. 8vo. pp. 171.

19. Researches into the Physical History of Mankind. By J. C. Prichard, M.D. F.R.S. M.R.I.A. &c. Third Edition. Vol. I.—London, 1836. 8vo. pp. 376; seven Plates.

20. Cyclopædia of Anatomy. Parts VII. and VIII. (Cilia—Death.)—London, 1836.

21. An Essay on the Operation for Cleft Palate. By George Bushe.—New York, 1835. 4to.

22. Valedictory Address delivered to the Graduates of the Jefferson Medical College. By G. M'Clellan, M.D.—Philadelphia, 1836. 8vo.

23. Coup d'Œil sur les Hopitaux de Londres, et sur l'Etat actuel de la Médecine et de la Chirurgie en Angleterre. Par Edwin Lee, Membre du College Royal des Chirurgiens à Londres.—Paris, 1836. 8vo. pp. 40.

24. De Aneurismatibus Arteriarum Cerebri. Auctore A. A. A. Stumpff.—4to. pp. 36. Berolini, 1836.

25. De Corneis Humani Corporis Excrescentiis. Auct. R. Fawsett Ainsworth, Mancuniensi.—Berolini, 1836. 4to. pp. 26.

26. Beobachtung einer auffallenden bisher unerkannten Structur des Seelenorgans bei Menschen und thieren. Von C. G. Eherenberg. Mit sechs Kupfertafeln. —Berlin, 1836. Fol. pp. 57.

27. Die Rein-chirurgischen Operationen des Geburthelfers. Von Dr. H. F. Kilian.—Bonn, 1835. 8vo. pp. 410.

27. Sammlung auserlesener Abhandlungen und Beobactungen aus dem gebiete der Ohrenheilkunde. Herausgegeben von Dr. C. G. Lincke. I.-II. Sammlung.—Leipzig, 1836. 8vo.

28. Handbuch der Physiologie des Menschen für Vorlesungen. Von Dr. Johannes Müller. Erster Band.—8vo. pp. 856. Coblentz, 1835.

29. Parallèle des divers Moyens de traiter les Calculeux. Par le Docteur Civiale.—Paris, 1836. 8vo. pp. 492.

30. Traité Pratique de la Syphilis. Par le Baron Philippe Boyer.—Paris, 1836. 8vo. pp. 284.

31. Anatomie du Système Dentaire, considérée dans l'Homme et les Animaux. Par Ph. Fr. Blandin, Chirurgien de l'Hôtel Dieu, &c.—Paris, 1836. 8vo. pp. 234.

32. Le Système Lymphatique, considérée sous les Rapports Anatomique, Physiologique, et Pathologique. Par G. Breschet, M.D. &c.—Paris, 1836. 8vo. pp. 304. iv. Planches.

33. Eine auserlesene Sammlung der nöthigsten abbildungen äusserlich sichtbaren Krankheitsformen, &c. Zum gebrauch für Praktische Chirurgie. Herausgegeben von Dr. A. Froriep.—4to. Weimar, 1836.

34. The Western Journal of the Medical and Physical Sciences. Edited by Daniel Drake, M.D., Professor of Medicine in Cincinnati College, and W. Wood, M.D.—Cincinnati, Ohio. Vol. III. No. 3. Jan. 1836.

35. The Jamaica Physical Journal. Edited by W. Arnold, M.D.—Kingston, Jamaica. January—April, 1836.

36. The India Journal of Medical and Physical Science. Edited by F. Corbyn, Esq.—Calcutta, January, February, and March, 1836.

37. Jahrbücher der In- und Ausländischen gesammten Medicin, Herausgegeben von C. C. Schmidt, M.D. &c.—Leipzig. No. I.—VI. 1836.

38. Handbuch derMenschlichen Anatomie. Von C. Fr. Th. Krause, M.D., Professor der Anatomie, &c. — Hanover, 1836. 8vo.

39. Darstellungen und Ansichten zur Vergleichung der Medicin in Frankreich, England, und Deutschland. Von Dr. Adolph Mühry.—Hanover, 1836. 8vo. pp. 283.

40. Manuel de Médecine Operatoire. Par J. F. Malgaigne, M.D., Prof., &c. 2nd Edit.—Paris, 1837. 12mo. pp. 773.

### INDEX TO VOL. II.

OF THE

### BRITISH AND FOREIGN MEDICAL REVIEW.

---

PAGE

A.

ABORTION, effects of ergot in . 276
Abousson, M. on worms in the air-passages . . . . 249
Abscesses, secondary, Mr. Travers on 10
Absorption, Dr. Carson on . . 599
Acne, M. Rayer on . . 150
Aconitine, Dr. Turnbull on . 500
Air-passages, worms in, death from 249
Alexander, Dr. on puerperal fever . 490
Albers, Dr. on aneurism of the thoracic duct . . . . 535
Alessandrini, Prof. on the development of muscle . . . 543
Anatomy, Comparative, Mr. Swan on 192
of parts concerned in operations . . 196
teachers of, regulations concerning . . 610
Aneurism of the thoracic duct . 535
of the heart, circumscribed 460
, popliteal, Mr. Adams on 600
Aphthæ of children, Dr. Naumann on 367
Asphyxia of children, M. Mende on 359
, Mr. Taylor on . . 422
Association, British, Report of . 594
Provincial Medical, 300, 601
Eastern branch 300
Southern branch, 606
Asthma, thymic, Dr. Hirsch on . 237
Atrophy of the heart, M. Bouillaud on . . . . . 336
Dr. Carswell on . . 460
Auscultation, Dr. Latham on . 480
Austria, vaccination in . . 280

B.

Becker, Dr. on the temperature of the sea . . . . . 520
Bell, Sir C. on the relations of nerves 215
Belladonna, its use in stricture and spasm . . . . . 261
Berndt, Dr. his clinical communications . . . . . 176
Beaumont, Dr. his experiments on digestion . . . . . 375

PAGE

Blizard, Sir Wm., Memoir of, by Mr. Cooke . . . . 205
Bloodletting, Dr. Wardrop on . 171
Blood in the heart, Dr. Wardrop on 218
catamenial, acidity of . 274
Books received for review . 306, 613
Botanical Society of Edinburgh . 611
Bouillaud, M. his Treatise on the Heart . . . 309
Bostock, Dr. analysis of calcareous tumours by . . . 164
Boiling, effects of, on meat and vegetables . . . 282
Bone, on the morbid anatomy of . 535
Brain, Dr. Philip on affections of the 129
structure of, Dr. Macartney on ib.
on the treatment of the diseases of . . 596
relation of, to the stomach . 370
Dr. Harlan on the functions of 518
Bright, Dr. on morbid changes in the peritoneum . . . 167
Brewster, Sir D. on the crystalline lens . . . 218, 599
Breschet, M. on the structure of the skin . . . . . 429
Brandis, Dr. on the use of cold in disease . . . . 529
British Association, report of . 594
Bullæ, M. Rayer on . . 149
Bushe, Mr. on the operation for cleft palate . . . . 493

C.

Cachexia, Mr. Travers on . 19
Cartilages of joints, on ulceration of 163
Catamenia, acidity of . . 274
Carditis, M. Bouillaud on . . 330
Cavities of the heart, changed dimensions of . . . 338
Carswell, Dr. his Pathological Illustrations . . . . 456
Cantharides, on the nature and use of 548
Carmichael, Mr. on the nature of tubercles . . . 597
Carson, Dr. on absorption . 599

PAGE
Calculous diseases, M. Civiale on . 276
Cæsarean operation, cases of 270, 568
Cellular membrane, erysipelas of . 23
Cholera in Italy, statistics of . 279
Children's Diseases, treatises on . 353
diagnosis of 353, 356
anatomical peculiarities of 354
dietetic rules for . . 386
evil of overtasking minds of ib.
on softening of the mucous membrane in . . 551
Chloride of soda in typhus fever . 64
intermittent fever 553
Chemistry, Mitscherlich's Elements of 188
Chelius, Professor, Surgical Observations by . . . . 259
Chalybeate water, artificial, formula for . . . . . 555
Chevallier, M. on colica pictonum . 573
Chomel, M. his clinical lectures on typhus . . . . . 33
Cicatrices, on warty tumours in . 161
Cinnabar fumigations, new mode of applying . . . . . 555
Circulation through the lungs, on the 518
Circulation in insects, on the . 213
Civiale, M. on calculous diseases . 276
Clinical medicine, Dr. Latham on . 473
Cleft palate, operation for, by Mr. Bushe . . . . . 493
new instruments for . 496
Constitutional irritation, definition of 3
Collins, Dr. his treatise on Midwifery 71
Convulsions, puerperal, Dr. Collins on 86
Cock, Mr. on malformations of the internal ear . . . . 167
Cooke, Mr. his memoir of Sir Wm. Blizard . . . . . 205
Coulson, Mr. on disease of the hip-joint . . . . . 231
Conjunctiva, on the arteries of the . 235
state of, in purulent ophthalmia . 505
Copaiba, new mode of administering 243
Compression, effect of, in orchitis . 253
Cornea, on foreign bodies in the . 269
Copenhagen, university and medical establishments of . . . 285
Conjunctivitis, Mr. Middlemore on 345
Combe, Dr. on the physiology of Digestion . . . . . 368
Contusions, their import in legal medicine . . . . . 415
Cold, on the use of, in diseases . 529
Colica pictonum, on the causes and cure of . . . . . 573
Colleges, medical, in the United States 583
Coroner's inquest, evidence in . 608
Crises in typhoid fever, M. Chomel on 41
Croup, spasmodic, Dr. Ley on . 112
from enlarged thymus 237
Creosote, on the medical properties of 168
simpler mode of obtaining 283

PAGE
Crustacea, on metamorphoses in . 214
Croton oil in affections of larynx . 584

D.

Denmark, present state of medicine in 285
state of the medical profession in . . 295
Devergie, Dr. on Forensic Medicine 390
Death, apparent, Mr. Taylor on . 425
Dermis, anatomy of the . . 431
Delphinia, Dr. Turnbull on . 500
Denman, Dr. his aphorisms on parturition . . . . 523
Decidua, on a new structure in the 600
Diabetes, Dr. Berndt on . . 179
sugar found in the blood in ib.
Digestion, on the physiology of . 368
Dietetics, Dr. Combe on . . ib.
Dictionary of medical terms, Dr. Palmer's . . . 508
Donné, Dr. on the pulse and respiration . . . . . 246
Dropsies, Dr. Osborne on . . 222
Dresden Lying-in Hospital, report of 273
Drake, Dr. biographical notice of . 305
Drinks, dietetic rules concerning . 387
Drowning, death by, Mr. Taylor on 426
Droste on softening of the mucous membrane . . . 551
Dupuytren, M. his new method of lithotomy . . . 99
Duparcque, M. on diseases of the uterus . . . . 511
Dunglison, Dr. on the medical institutions of America . . 583
Dublin Lying-in Hospital, statistics of 72

E.

Ear, internal, malformation of . 167
Eastern Provincial Medical Association . . . . . 300
Ecthyma, M. Rayer on . . 154
Elliottson, Dr. on creosote . . 168
Endocarditis, nature and symptoms of 321, 326
Entozoa, tubercle and cancer classed as 598
Epilepsy, on the use of indigo in . 244
Epidermis, anatomy of . 429, 446
Erysipelas, Mr. Travers on . 23
pathology and treatment of . . 25-6
its connexion with puerperal fever . 487
Eruption, peculiar, in typhoid fever, 36, 40
Ergot, its use in abortion . . 276
parturition . 525
Erectile tumours, surgical treatment of 564
Ether, on the production of . 191
Examiners of Apothecaries' Company, report of . . . 606
Eye, natural cures of diseases of . 270
Mr. Middlemore on diseases of 342

PAGE

F.

Fever, puerperal, Dr. Collins on . 91
Mr. Moore on . 481
Dr. Alexander on 490
nature of . 482
from morbid state of the blood . 483
its connexion with erysipelas . 487
importance of discrimination in . 491
typhoid, nature of . 59
M. Chomel on . 33
symptoms and progress of . 34
appearance after death in . 43
exciting causes of . 51
diagnosis of . 55
prognosis of . 56
treatment of . 59
of growth, Dr. Reich on the 219
intermittent, use of chloride of soda in . . . 553
treatment of . 176
Fistulæ, vesico-vaginal, new mode of treating . . . . . 561
intestinal, use of actual cautery in . . . 567
Fletcher, Dr. biographical notice of 302
Fœtus, diseases of the . . 365
position of the head of, in labour . . . 524
without heart or lungs, account of a . . 596
length of, at different periods 406
Follicles, intestinal, diseased in typhus 45
Forceps, use of, in delivery . 76
Formulary of new remedies, Magendie's, 207
hospitals, Edwards, &c. on, 210
Fothergillian medal for 1837-8 . 302
Forensic medicine, Devergie and Taylor on . . . 390
Food, proper kinds and quantity of 383
Fracture of the neck of the femur . 159
radius, M. Goyraud on 256
Fricke, Dr. on compression in orchitis 253
France, medico-legal proceedings in 392
Fumigations of cinnabar, new method of . . . . . 555

G.

Gangrene of the lungs in the insane 546
Gangrenous inflammation, Mr. Travers on . . . . . 28
Gas, cure of hydrocele by . . 267
Geneva, on suicide in . . 277
Gerdy, M. on the morbid anatomy of bone . . . . 535
on the treatment of hernia 566
Glands, sudorific, account of . 438
lymphatic, inflammation of 558
Glanders in man, cases of . . 241
Glaucoma, Mr. Middlemore on . 348

PAGE

Glosso-pharyngeal nerve, functions of 600
Gonorrhœa, analogy to ophthalmia . 504
Goyraud, M. on fracture of the radius 256
Gout, the magnet a remedy in . 246
Gully, Dr. his translation of Magendie's Formulary . . . 207
Guislain, M. on gangrene of the lungs 546
Guy's Hospital Reports . . 198
Guyot, M. on the effect of heat on wounds . . . . 263
Gurlt, M. on the structure of the skin 429

H.

Hawkins, Mr. on warty cicatrices . 161
Hanging, death from . . 418
Harlan, Dr. his medical researches 518
Heart, mechanism of the . . 599
change of the cavities of . 338
polypi of . . . 339
causes of the sounds of . 598
diseases of, M. Bouillaud on 309
causes of . 311
progress of . 312
prognosis of . 313
use of veratria in 499
atrophy of . . 336
neuroses of . . . 336
neuralgia of . . 337
Heat, effect of, on wounds . . 263
Hecker, Dr. on the medical societies of Prussia . . . 577
Hemorrhage, uterine, during labour 81
fatal, from uterine tumour . . 571
Herpes, Mr. Rayer on . . 149
Hernia, use of belladonna in . 261
on the treatment of . 566
Henry, Dr. obituary of . . 612
Hip-joint, Mr. Coulson on disease of 231
Hirsch, Dr. on thymic asthma . 237
Hooping-cough, endermic treatment of, 178
Howship, Mr. on fracture of the neck of the femur . . . 159
Hospital Reports, Guy's and St. Thomas's . . . 198
Hospitals of Ireland, account of . 185
Hosack, Dr. biographical notice of . 306
Hourmann, M. on the pneumonia of the old . . . . 543
Houston, Dr. on a fœtus without heart or lungs . . . . 596
Hufeland, Professor, obituary of . 613
Hydrocele, cure of, by gaseous injections . . . . 267
Hydropericardium, M. Bouillaud on 331
Hypertrophy, Dr. Carswell on . 456
of the heart, M. Bouillaud on . . 332

I.

Illustrations of the forms of disease, Dr. Carswell's . . . 456
Impetigo, M. Rayer on . . 152
Infants, on the care of . . 221

5

PAGE

Infants, anatomical characters of . 406
Infanticide, M. Divergie on . 405
from obstruction of the air-passages . 414
Indigo, its use in epilepsy . . 244
Induration of cellular membrane in children . . . . . 261
Inflammation, gangrenous, Mr. Travers on . . . . . 28
of lymphatics, M. Velpeau on . . 556
Inhalent apparatus of the skin . 440
Insane, gangrene of the lungs in the 546
Incontinence of urine, treatment of 555
Iritis, Mr. Middlemore on . . 347
Irritation, constitutional, Mr. Travers on . . . . . 1
Italy, statistics of the cholera in . 279

J.

Jacksonian prize for 1835 . . 302
Jobert, M. on vesico-vaginal fistulæ 561
Journals, Foreign Medical, account of 231
Jüngken on purulent ophthalmia . 501
Jurisprudence, Medical, treatises on 390
an improper term 422

K.

Key, Mr. on ulceration . . 166
Kidneys, cases of disease of the . 226
Kilian, Professor, on tumour of the uterus . . . . 571

L.

Latham, Dr. on clinical medicine . 473
Larynx, on croton oil in affections of 554
Lallemand, M. on the cure of erectile tumours . . . . . 564
Latin, on the study of, by apothecaries 607
Lactic acid, Magendie on . . 208
Laryngismus stridulus, Dr. Ley on 112
history of . 114
causes of . 116
pathology of 121
treatment of 125
Lebaudy, Dr. his anatomy of the regions, &c. . . . 196
Legal medicine, definitions of . 391
treatises on . 390
Leeches to the cervix uteri, use of . 516
Leucorrhœa, history of . . 553
Lead, new treatment of diseases from 576
Lectures in the United States . 594
Lens, crystalline, on the structure of 218
dislocation of, Mr. Middlemore on . . . . . 350
Letters to a Mother, on the care of children . . : 221
Ley, Dr. on laryngismus stridulus . 112
Lichen, M. Rayer on . . 154
Lithotomy, Dupuytren's new method of . . . . . 98
statistics of . . 105
causes of failure in France 106

PAGE

Lithotomy and lithotrity compared 466
Lithotrity and lithotomy compared ib.
Lisfranc on white swellings . 266
Lisfranc, M. on the treatment of white swellings . . . 567
Löwenhardt, M. on Oophoritis . 526
Lungs, gangrene of, in the insane . 546
Lungs, state of, in infants . . 410
old persons, in pneumonia . 544
Lymphatics, on the structure of . 442
diseases of . 556

M.

Macartney, Dr. on the structure of the brain . . . 129
Mayo, Mr. on ulceration of cartilage 163
Macilwain, Mr. on unity of the body 180
Magnet, the, a remedy for gout . 246
Magendie, M. his formulary of new medicines . . . 207
Metastasis, Mr. Travers on . 8
Mercurial cachexia, case of . 21
Membranes, reflected, erysipelas of 24
Medico-chirurgical Transactions . 158
Medical charities of Ireland, Mr. Phelan on . . . 183
Medicine, state of in Denmark . 285
Meals, proper periods of . 380
Medicine, legal, treatises on . 390
clinical, Dr. Latham on 473
Medical witnesses' bill . . 608
Meyer, Dr. on the cæsarean section 568
Midwifery, Dr. Collin's treatise on 71
Mitcherlich, his elements of chemistry 188
Middlemore, Mr. on diseases of the eye 342
Moore, Mr. on puerperal fever . 481
Mucific apparatus of the skin . 445
Muscles, influence of the nerves in developing . . . 543

N.

Nature, her powers, in curing diseases of the eye . . . 270
Nervous system, Dr. Philip on the physiology of . . . 131
Nerves, on the relations of . 215
Neuroses and neuralgia of the heart 386
Nerves, Dr. Reid on the functions of 600
of the eyeball, Mr. Walker on ib.
Nitrate of silver, discoloration from 156
Nux vomica, in incontinence of urine 555

O.

Obituary . . . 302-612
Osborne, Dr. on dropsy . 222
Otto, Dr. on rheumatism . 252
the state of medicine in Denmark . . 285
Ophthalmia, traumatic, Dr. Schindler on 267
Mr. Midlemore on . 347
purulent Drs. Jüngken and Tschetirkin on 501
causes of 508

PAGE
Opium, new principles in . 207
Oxalic acid, on the action of . 284
Oophoritis, Dr. Lowenhardt on . 526
Ovaria, inflammation of . ib.
Osteitis, M. Gerdy on . . 535
O'Beirne, Dr. on tetanus . 595
Obstetrician's vade mecum . 528
duties in the lying-in room . . 524

P.

Palate, cleft, operation for . 493
Palmer, Dr. his dictionary of medical terms . . . 508
Papillæ of the skin, anatomy of 432
Perforation of the head in parturition 77
intestines by worms 519
Peritoneum, on morbid changes in the 167
Perspiration, suppressed, relation to dropsy . . . 222
Petrifaction of animal bodies . 532
Pericarditis, M. Bouillaud on . 315
acute, diagnosis of 316
chronic . . 319
treatment of 320
Phelan, Mr. on the charities of Ireland 183
Philosophical Transactions for 1835, 212
Philip, Dr. on affections of the brain 129
Plates, anatomical, effects of on students 197
Plates, M. Rayer's, account of . 157
Plates in this volume, explanation of 615
Pneumonia in old persons . . 543
Poisons, effects of on vegetables . 518
Porrigo, M. Rayer on . . 152
Polypi of the heart, M. Bouillaud on 339
Polypus, bronchial, expectoration of the heart . . . 554
Poem, a medical, by Professor Bang 301
Presentations, kinds of in labour 78
Prichard, Dr. on the treatment of diseases of the brain . . 596
Puerperal fever, Dr. Collins on 91
Mr. Moore on 481
Dr. Alexander on 490
convulsions, Dr. Collins on 86
Pulse, its relation to temperature and respiration . . . 246
Putrefaction, in relation to legal medicine . . . 396
varieties and stages of 397-400
Pregnancy, new diagnostic sign of 275
Prussia, medical societies of . 577
revaccination in the army of 280
Pregnancy, signs of . . 402
duration of . . 403
extra uterine . 570
Pneumonia in old persons . . 543
Provincial medical association, report of . . . 601
meeting of 300
eastern branch of ib.
southern branch of 606

PAGE

R.

Rayer, M. on diseases of the skin 147
Radius, on fractures of the . 256
Ranunculaceæ, Dr. Turnbull on 499
Respiration, Dr. Stevens on . 216
its relation to the pulse and temperature . 246
Reich, Dr. on the fever of growth 219
Revaccination in the Prussian army 280
Reid, Dr. on the functions of the nerves 600
Registration of births, deaths, and marriages, act for . . 611
Rhumatism, treatment of, by carburet of sulphur . . . 252
Romer, Dr. on the arteries of the cornea 235
Roussel de Vauzemâ on the structure of the skin . . . 429
Rupture of the uterus, Dr. Collins on 87
Ryan, Dr. his translation of Edwards' formulary . . . 210
Denman's aphorisms by 523

S.

Sea on coasts, temperature of . 520
Segato, Sig. on animal petrifaction 532
Scabies, M. Rayer on . . 149
Scarlatina, Dr. Berndt on . 178
Schultz, M. on the physiology of vomiting . . . 537
Skin, inhalent apparatus of . 440
mucific apparatus of . 445
colorific apparatus of 445-451
erysipelas of . . 23
hereditary relaxation of . 568
on diseases of, by M. Rayer 147
on the structure of . 429
sudorific apparatus of . 437
Softening of the mucous membrane in children . . . 551
Societies, medical, in Prussia . 577
Sounds of the heart, on the causes of the . . . 598
Small-pox, salutary effects of . 250
Sneezing, case of, cured by snuff . 245
Squamous diseases, M. Rayer on 155
Stricture of the urethra, on belladonna in . . . 261
Statistics of calculous diseases . 276
Strangulation, death from . 419
Stomach and brain, relation of . 370
singular case of perforation of 375
relative shape of, in different ages . . . 539
Stone, new method for the, by Dupuytren . . . 98
St. Thomas's hospital, reports of 198
Strophulus, M. Rayer on . 154
Surgery, Walther's system of . 202
arrangement of the subjects of 203
Surgical observations, by Chelius 259
Sugar in the blood of diabetics . 281
Sudorific apparatus of the skin . 437
Superfœtation, remarks on . 405
Suicide in Geneva, statistics of . 277

PAGE

Swan, Mr. his illustrations of the nerves 192
Sympathy, physiology of . 139
Sycosis, M. Rayer on . . 151
Sympathies, classification of . 181

T.

Taylor, Mr. on forensic medicine 390
Temperature of fishes, Dr. Davy on 217
the sea, Dr. Becher on 520
Temperature, its relation to the pulse and respiration . . . 246
Testicle, inflammation of, new mode of treating . . . 253
Teeth, on the treatment of . 373
Tetanus, Dr. O'Beirne on . 595
Thigh-bone, how retained in its place 236
Thymus gland, a cause of croup . 237
Thoracic duct, aneurism of . 535
Travers, Mr. on constitutional irritation 1
Trismus nascentium, Dr. Collins on 97
Transactions of the Med. Chir. Society 158
Tscheterkin, Dr. on purulent ophthalmia 501
Tubercular inflammations, M. Rayer on 155
Tubercles, on the deposition of 12
the nature of . . 597
Tumours of the uterus, calcareous 164
Turnbull, Dr. on the ranunculaceæ 494
Twins, Siamese, account of . 294
Typhoid fever, M. Chomel on . 33
nature of . 59
varieties of . 53
symptoms and progress of . 34
appearances after death in 43
principal organs affected in 48
exciting causes of . 51
on the contagion of . 52
diagnosis of . . 55
prognosis of . . 56
treatment of . 59
Tyrrell, Mr. on the circulation in insects . . . 213
Typhus, carbuncular, from glanders 242
Tympanites, on the treatment of . 552

PAGE

U.

Ulcers in the intestines in fever 44
cicatrization of 45
University of Edinburgh, prizes by 609
Unity of the body, Mr. Macilwain on 180
United States, medical institutions of the . . . 583
Urine, coagulable in dropsy . 323
retention of, use of belladonna in 261
Uterinus vagitus . . 409
rupture of, Dr. Collins on 87
calcareous tumours in . 164
Duparcque on diseases of the 511
hemorrhage, Duparcque on 515
fatal case of 571
Ulceration, Mr. Key on . 166

V.

Valve, bicuspid, Mr. King on the 216
Vaccination in France, report on 251
Austria . . 280
Vaccine veins, experiments on . 251
Vagitus uterinus . . 409
Veratria, Dr. Turnbull on . 499
Voice, human, on the tones of the 214
Vomiting, physiology of . . 587

W.

Walther, his system of surgery 202
Walshman, Dr. obituary of . 305
Watering places, picture of . 372
Weber, Dr. on the mechanism of the thigh-bone . . . 236
Wendt, Dr. on the structure of the skin 429
White swellings, on the treatment of 266-567
White lead, workers in, diseases of 573
Worms in the air-passages, death from 249
Wounds, effect of heat on . 263
relation of, to legal medicine 427
Women, on the critical age in . 513

Z.

Zoology, new magazine of . 301

END OF VOL II.

PRINTED BY J. AND C. ADLARD,

BARTHOLOMEW CLOSE.

